# Importance of Defining Anatomy: Uncovering an Unexpected Finding With CT Coronary Angiography After Abnormal SPECT Results

**DOI:** 10.1002/ccr3.71267

**Published:** 2025-11-21

**Authors:** Anushree Puttur, Laith Alhuneafat, Omar Obeidat, Andreas Kyvernitakis, Ahmad Jabri, Abdallah Naser, Ahmad Al‐abdouh, Farhan Katchi

**Affiliations:** ^1^ Department of Medicine Allegheny Health Network Pittsburgh Pennsylvania USA; ^2^ Department of Cardiovascular Disease University of Minnesota Minneapolis Minnesota USA; ^3^ Graduate Medical Education University of Central Florida College of Medicine Orlando Florida USA; ^4^ Internal Medicine Residency Program HCA Florida North Florida Hospital Gainesville Florida USA; ^5^ Department of Cardiology UnityPoint Health Cedar Rapids Iowa USA; ^6^ Heart and Vascular Center, Henry Ford Hospital Detroit Michigan USA; ^7^ Division of Hospital Medicine University of Kentucky Lexington Kentucky USA; ^8^ Cardiovascular Division Washington University School of Medicine St. Louis Missouri USA

**Keywords:** acute aortic syndrome, acute chest pain, computed tomography, coronary artery disease, myocardial infarction

## Abstract

Coronary CT angiography extends beyond detecting obstructive coronary artery disease by providing vital anatomical insights into extra‐coronary pathologies, including acute aortic syndromes. This non‐invasive modality enables early identification of life‐threatening conditions and informs multidisciplinary management strategies, underscoring its critical role in comprehensive diagnostic evaluation and patient‐centered care.

## 1 Introduction

Acute aortic syndrome (AAS) is a life‐threatening condition that conventionally includes classic aortic dissection, penetrating atherosclerotic ulcer, and intramural hematoma [1]. With the improvement in temporal and spatial resolution in CT imaging, aortic abnormalities can be more efficiently diagnosed [2]. This case highlights how anatomical assessment using CCTA can guide more appropriate management in select patients. Relying solely on catheterization after an abnormal stress test may not always be the best choice. In our patient, that approach could have delayed care and led to serious complications.

## 2 History of Presentation

A 75‐year‐old male with a past medical history of hypertension, hyperlipidemia, and recurrent nephrolithiasis presents to the emergency department with one week of flank pain that radiated to his upper back and chest wall. His pain, initially described as similar to prior episodes of nephrolithiasis, was episodic, not aggravated on exertion, and not associated with shortness of breath or diaphoresis. On arrival, he was afebrile with a heart rate of 89 beats/min, a respiratory rate of 15 breaths/min, a blood pressure of 169/77 mmHg, and an oxygen saturation of 96% on room air. Physical examination was largely unremarkable, with no abdominal or costovertebral angle tenderness, adventitious breath sounds, murmurs, rubs, or gallops.

## 3 Differential Diagnosis

His initial differential diagnosis included nephrolithiasis, pyelonephritis, acute coronary syndrome, pancreatitis, cholecystitis, choledocholithiasis, and acute aortic dissection.

## 4 Investigations

His laboratory results included negative cardiac enzymes and an ECG without acute ischemic changes. A non‐contrast CT of the abdomen and pelvis was obtained to evaluate for renal colic. It showed triple vessel coronary artery disease and annular calcifications in the abdominal aorta. However, no acute inflammation, nephrolithiasis, or hydronephrosis was seen. On outpatient follow‐up, he underwent an exercise single‐photon emission computerized tomography (SPECT) imaging for further investigation of his prior imaging findings on which he earned a Duke Treadmill score of 3.5 and 6.9 METS. Exercise ECG showed inferolateral ST‐segment depressions suggestive of ischemia. SPECT revealed a small fixed defect in the inferior and apical regions, but no inducible ischemia.

## 5 Management (Medical/Interventions)

**FIGURE 1:**
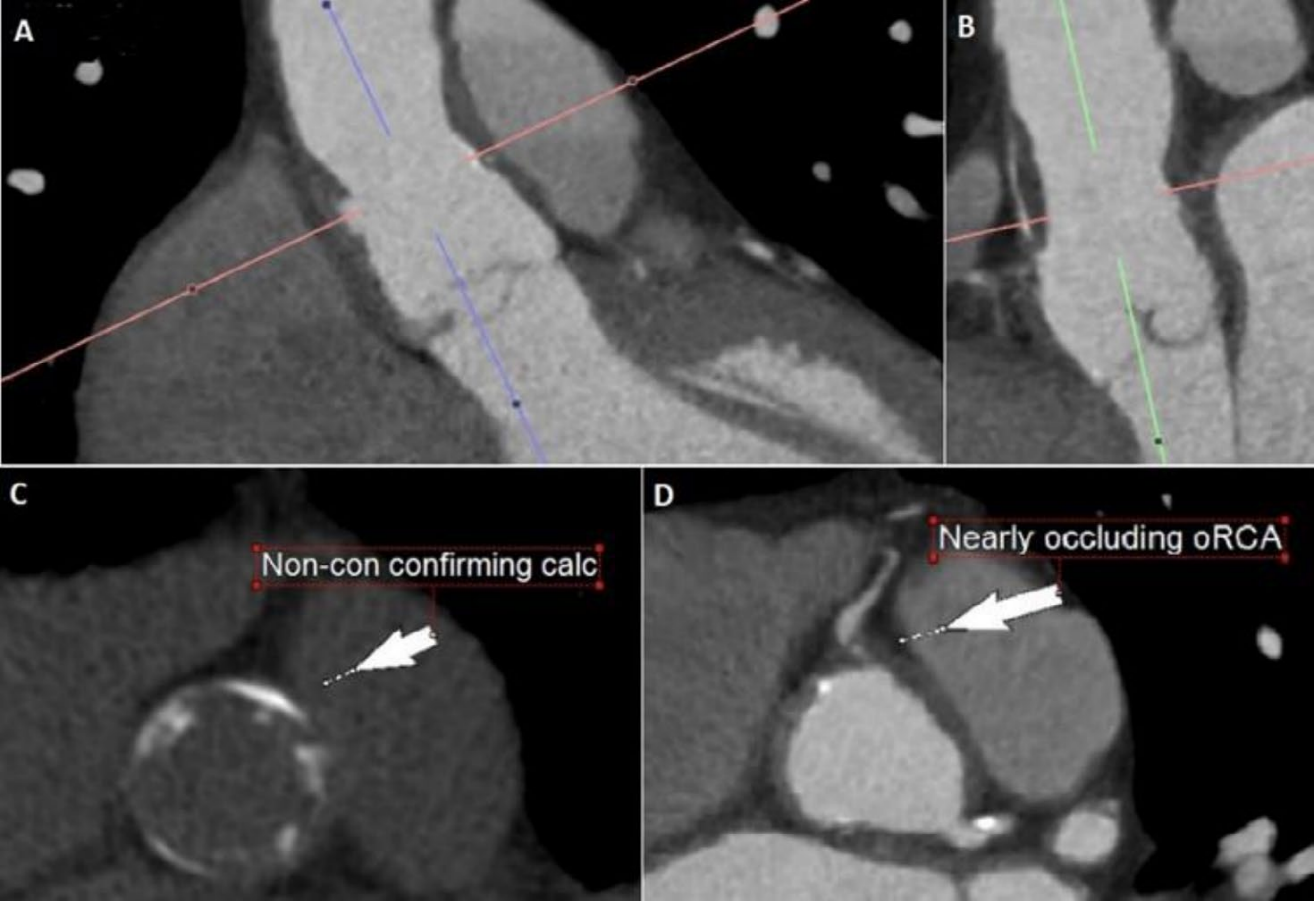
Computed tomography coronary angiography revealing two separate small penetrating aortic ulcerations in the aortic root (A, B) and associated severe burden of atherosclerosis in the aortic wall (C), with subsequent compression and near‐occlusion of the ostium of the right coronary artery (oRCA) (D).

Due to the atypical nature of his symptoms and the patient's apprehension towards pursuing further invasive diagnostic testing, the decision was made to proceed with a prospectively gated, contrast‐enhanced, dual‐source 64‐slice coronary CT angiogram. CT coronary angiography revealed two small penetrating aortic ulcers in the aortic root (Figure 1A,B), extending into the proximal ascending aorta. There was also severe atherosclerotic burden in the aortic wall (Figure 1C). There was evidence of evolution of a penetrating aortic ulcer into an intramural hematoma with subsequent compression and near‐occlusion of the ostium of the right coronary artery (oRCA) (Figure 1D). After multidisciplinary discussion, the plan was to proceed with surgical intervention given the potential morbidity/mortality risk of progressive or acute aortic compromise. The surgical plan included aortic root replacement with a valve‐sparing procedure and coronary artery bypass grafting for the right coronary artery obstruction.

## 6 Discussion: Association With Current Guidelines/Position Papers/Current Practice

AAS, a spectrum of life‐threatening conditions with similar underlying pathophysiology, conventionally includes classic aortic dissection, penetrating atherosclerotic ulcers, and intramural hematomas. These syndromes overlap, are dynamic, and require rapid and accurate diagnosis for effective management [1]. Classic aortic dissection is caused by an intimal tear, while intramural hematoma occurs without a tear due to the rupture of the aortic vasa vasorum or in the presence of a penetrating aortic ulcer. The intramural hematoma can extend longitudinally through the medial layer, weakening the aorta and eventually causing either outward rupture of the aortic wall [3]. Three imaging modalities are commonly used to diagnose acute aortic syndromes: CT angiography (CTA), magnetic resonance angiography (MRA), and transesophageal echocardiography (TEE) [4].

CTA is widely considered the first‐line modality for evaluating suspected acute aortic syndromes due to its speed, availability, and high diagnostic accuracy, with sensitivity and specificity approaching 98%–100%. It also offers the advantage of identifying alternative thoracic pathologies, such as pulmonary embolism or pneumonia, during the same scan [4]. MRA provides excellent soft tissue contrast and avoids ionizing radiation, making it suitable for follow‐up imaging or for patients who cannot receive iodinated contrast. However, its longer acquisition time and limited accessibility often preclude its use in unstable or emergent cases [3, 4]. TEE, by contrast, can be performed at the bedside and is particularly useful in unstable patients. It provides high‐resolution imaging of the proximal aorta and aortic valve, though it may have limited visualization of the distal aorta and is subject to operator dependency. While TEE has high sensitivity for aortic dissection, false‐positive findings can occur, particularly in the ascending aorta [3, 4].

Aortic, pulmonary, and coronary pathologies often present with similar clinical signs and symptoms. As such, several studies have discussed the utility and feasibility of “triple rule‐out” (TRO) CT, a prospectively ECG‐gated, contrast‐enhanced coronary CT angiography protocol that enables simultaneous evaluation of three critical causes of acute chest pain: acute coronary syndrome, pulmonary embolism, and aortic dissection [5]. It is particularly useful in intermediate‐risk patients with nondiagnostic initial testing, offering a comprehensive and efficient diagnostic strategy in the emergency setting [5]. TRO‐CT has demonstrated a high negative predictive value (approaching 99%) in ruling out life‐threatening cardiopulmonary events, helping avoid unnecessary invasive procedures and expediting safe discharge when negative. Additionally, it can detect alternative pathologies, such as pneumonia or esophageal disease, in up to 10%–15% of cases [5, 6]. However, this technique requires careful protocol design, optimized contrast timing, and expert interpretation, as it often involves higher radiation exposure and greater technical complexity compared to single‐system scans [5, 6]. With the improvement in temporal and spatial resolution in CT imaging, aortic abnormalities can be more efficiently diagnosed [2].

Current guidelines recommend functional or anatomic evaluation for possible cardiac chest pain based on various factors [7]. Recent studies have shown improved risk stratification by combining functional ischemia testing with SPECT and anatomic assessment with CCTA. In a study by van Werkhoven et al., CCTA improved risk stratification in patients with > 50% stenosis, regardless of prior SPECT findings, indicating an additive benefit [8]. In our case, CCTA performed after functional testing uncovered a critical and unexpected finding. This emphasizes the complementary benefits of functional and anatomical assessment in evaluating chest pain. Notably, many professional societies now endorse CCTA as a first‐line diagnostic modality in patients with stable chest pain and intermediate risk [9, 10]. Additionally, its utility also extends to defining congenital coronary anomalies, particularly when invasive angiography is inconclusive [9, 10].

Our patient had acute aortic syndrome in Stanford A distribution and an intramural hematoma extending into the RCA. As lesions in the Stanford A distribution often require urgent open surgical management, CCTAs offer an efficient, cost‐effective, and minimally invasive method of diagnosis that can provide rapid diagnosis in these potentially life‐threatening scenarios [2]. Aortocoronary dissection, although rare, can occur with forceful administration of contrast, manipulation of the guidewire, catheter, or stent and can quickly lead to fatal consequences secondary to cardiac tamponade, coronary artery occlusion, or further propagation of the dissection into the descending aorta [11]. This risk may potentially be higher in our patient with an intramural hematoma in the RCA [12]. The increased risk of dissection in the setting of a preexisting intramural hematoma as well as the RCA being the affected artery in our case made early diagnosis and risk mitigation through CCTA an especially valuable tool.

In conclusion, this case highlights that coronary CT angiography is not only a valuable diagnostic tool in identifying obstructive coronary artery disease but also provides a valuable extra‐coronary assessment that may heavily influence patient management. In rare but important cases like this, non‐invasive imaging of the coronary, aortic, and pulmonary anatomy can reveal serious underlying disease. Invasive coronary angiography may miss the diagnosis and carry unnecessary risks.

This abstract has been previously published in a conference. See reference number [13].

## Author Contributions

**Anushree Puttur:** writing – original draft, writing – review and editing. **Laith Alhuneafat:** writing – original draft, writing – review and editing. **Omar Obeidat:** writing – original draft, writing – review and editing. **Andreas Kyvernitakis:** writing – original draft, writing – review and editing. **Ahmad Jabri:** writing – original draft, writing – review and editing. **Abdallah Naser:** writing – original draft, writing – review and editing. **Ahmad Al‐abdouh:** writing – original draft, writing – review and editing. **Farhan Katchi:** writing – original draft, writing – review and editing.

## Consent

Written informed consent was obtained from the patient to publish this case report.

## Conflicts of Interest

The authors declare no conflicts of interest.

## Data Availability

Data sharing is not applicable to this article as no datasets were generated or analyzed during the current study. All relevant clinical details are included within the manuscript.
